# Fibronectin is required for proper extracellular matrix organization and cardiac outflow tract elongation in *Xenopus laevis*

**DOI:** 10.3389/fcell.2026.1833711

**Published:** 2026-05-29

**Authors:** Javiera Jorquera, Isidora Sovino, Catalina Jara-Gonzalez, Ignacio Rosales, Paula G. Slater, Cecilia Arriagada

**Affiliations:** 1 Centro de Biología Celular y Biomedicina (CEBICEM), Facultad de Ciencias, Universidad San Sebastián, Santiago, Chile; 2 Departamento de Ciencias Biológicas y Químicas, Facultad de Ciencias, Universidad San Sebastián, Campus Los Leones, Santiago, Chile; 3 Laboratorio de Neuro-regeneración y metabolismo, Centro Científico y Tecnológico de Excelencia Ciencia & Vida, Fundación Ciencia & Vida, Santiago, Chile

**Keywords:** extracellular matrix, fibronectin, heart development, second heart field, *Xenopus*

## Abstract

**Introduction:**

Congenital heart defects frequently arise from alterations in cardiac outflow tract (OFT) elongation, a process that depends on the coordinated deployment of second heart field (SHF) progenitor cells and their interactions with the extracellular matrix (ECM). Among ECM components, fibronectin (Fn1) and tenascin-C (TnC) have emerged as key regulators of cardiac morphogenesis; however, the cellular organization of the SHF and the dynamics of its ECM environment remain poorly characterized in externally developing vertebrate models.

**Methods:**

Here, we investigated the cellular and extracellular architecture of SHF-associated cells localized to the dorsal pericardial wall (DPW) during heart development in Xenopus laevis using immunofluorescence, three-dimensional reconstructions, quantitative analyses, and functional depletion experiments.

**Results:**

We found that SHF-associated cells undergo a stage-dependent transition from a predominantly monolayered organization at NF35 to a multilayered structure at NF42, accompanied by dynamic ECM remodeling characterized by increased expression of Fn1, TnC, and collagen I (Col I), as well as redistribution of ECM components within the DPW. Functional depletion of Fn1 disrupted cardiac morphogenesis, resulting in OFT shortening and reduced ventricular size, while also decreasing TnC and Col I levels without affecting TnC spatial organization within the DPW.

**Conclusion:**

Together, these findings support a role for Fn1 in regulating ECM assembly within SHF-associated cells and demonstrate that ECM remodeling contributes to DPW organization during OFT elongation, highlighting Xenopus laevis as a valuable model for studying ECM-driven mechanisms of cardiac morphogenesis.

## Introduction

Congenital heart defects are among the most common developmental disorders in humans and, in many cases, arise from early alterations in the morphogenetic processes that shape the embryonic heart, particularly the elongation of the outflow tract (OFT) (Neeb et al., 2013; Tsao et al., 2022) and the formation of the cardiac chambers (Bruneau, 2008). Proper OFT elongation depends on the precise coordination of proliferation, migration, and organization of cardiac progenitor populations, as well as on dynamic interactions between cells and the surrounding extracellular matrix (ECM) (Buckingham et al., 2005; Silva et al., 2021).

One of the principal cellular lineages contributing to OFT elongation is the second heart field (SHF), a population of multipotent progenitor cells located adjacent to the developing heart that are progressively incorporated into the arterial and venous poles of the heart tube (Buckingham et al., 2005; Kelly et al., 2014). The ECM is a fundamental regulator of embryonic morphogenesis, as it not only provides structural support but also delivers biochemical and mechanical cues that influence cell polarity, migration, cell shape, and cell fate decisions (Hynes, 2009; Rozario and DeSimone, 2010). During cardiac development, ECM components are dynamically deposited and reorganized around cardiac progenitors, contributing to tissue integrity and to the transmission of forces necessary for cardiac tube elongation (Czirok et al., 2006; Le and Stainier, 2004). Despite important advances in identifying genetic regulators of cardiogenesis, the mechanisms by which the ECM contributes to tissue organization and cardiac progenitor behavior remain poorly understood.

Several ECM proteins, including fibronectin (Fn1), tenascin-C (TnC), collagen type I (Col I), and laminin, have been described as key regulators of heart formation (Silva et al., 2021). Among these, Fn1 and TnC have emerged as important regulators of cardiac morphogenesis. Fn1 is a large glycoprotein that assembles into fibrillar networks through integrin-dependent interactions and plays a central role in cell–ECM adhesion and in the organization of the tissue microenvironment (Hynes, 1986; Pankov and Yamada, 2002). Genetic studies in different vertebrate models have demonstrated that Fn1 is essential for developmental processes that require extensive tissue remodeling, such as gastrulation, somitogenesis, and cardiovascular organogenesis (Astrof et al., 2007a; Astrof et al., 2007b; Chen et al., 2015; George et al., 1993; Le and Stainier, 2004; Warkala et al., 2021).

In contrast, TnC is a hexameric ECM protein that is highly expressed during embryonic development and re-expressed in pathological contexts, such as tissue repair and cancer (Chiquet-Ehrismann et al., 1986; Chiquet-Ehrismann and Chiquet, 2003; Midwood et al., 2016). Unlike Fn1, TnC has been described as an anti-adhesive or adhesion-modulating molecule that regulates cell shape, tissue plasticity, and integrin-mediated signaling (Radwanska et al., 2017). During cardiac development, TnC is preferentially localized in regions undergoing intense morphogenetic remodeling, including the OFT and domains associated with the SHF (Imanaka-Yoshida et al., 2003; Imanaka-Yoshida and Aoki, 2014). Accumulating evidence indicates that Fn1 and TnC functionally interact within the ECM. *In vitro* studies have shown that TnC can associate with Fn1 fibrils and modulate integrin-mediated adhesion and signaling, thereby influencing the balance between cell adhesion and tissue plasticity (Chiquet-Ehrismann et al., 1988; Midwood et al., 2016; Orend et al., 2003; To and Midwood, 2010). In the SHF of *Mus musculus* (mouse) embryos, loss of Fn1 leads to mislocalization of TnC and disruption of ECM organization, which is associated with defects in OFT elongation and cardiac morphogenesis (Arriagada et al., 2025). These findings suggest that a finely regulated balance between adhesive and modulatory ECM components is crucial for maintaining SHF tissue architecture.

Studying cardiac development in mouse models can be technically challenging because it occurs *in utero*. Therefore, comparative studies across vertebrate models, particularly those with external embryonic development, are valuable for advancing our understanding of cardiac morphogenesis and for distinguishing conserved mechanisms from lineage-specific processes. *Xenopus laevis* has been widely used as a model organism for studying early vertebrate development due to its external development and ease of experimental manipulation. Moreover, *Xenopus* embryogenesis proceeds rapidly, enabling the study of the entire cardiovascular developmental process within a relatively short time frame (Carotenuto et al., 2023; Kaltenbrun et al., 2011). Although the *Xenopus* heart displays a simpler anatomy than that of mammals, cardiogenic signaling pathways, as well as the fundamental processes of cardiac tube formation, OFT elongation, and SHF progenitor contribution to OFT growth, are conserved (Brade et al., 2007; Kolker et al., 2000; Lee and Saint-Jeannet, 2011; Mohun et al., 2000; Sater and Jacobson, 1989; Schneider and Mercola, 2001). However, the organization of the SHF and the composition and dynamics of its ECM in *Xenopus* remain relatively poorly characterized.

In this study, we investigated the cellular and extracellular architecture of the SHF associated with the dorsal pericardial wall (DPW) during cardiac development in *Xenopus laevis*, with a particular focus on dynamic ECM remodeling. We characterized tissue organization, cell morphology, and epithelial features of SHF cells in the DPW at different developmental stages and analyzed the temporal and spatial distribution of key ECM components, including Fn1, TnC, Col I, and laminin. In addition, we assessed the potential role of Fn1 in OFT elongation and ECM organization, as well as its impact on TnC levels.

Together, our results provide new insights into how ECM remodeling contributes to SHF architecture and cardiac morphogenesis in a non-amniote vertebrate and reveal both conserved and divergent aspects of Fn1–TnC interactions during heart development. These findings support the use of *Xenopus laevis* as a valuable comparative model for investigating ECM-dependent mechanisms relevant to the origin of congenital heart defects.

## Materials and methods

### *Xenopus laevis* embryo breeding and maintenance

Adult female and male *Xenopus laevis* were pre-injected with 50 μL human chorionic gonadotropin (hCG, 1000 IU/mL), 72 h later they received a booster injection of 700 μL and 300 μL hCG (1000 IU/mL), respectively. Females were crossed with adult males by amplexus, and external fertilization occurred approximately 12 h post-injection. Fertilized eggs were collected and dejellied by incubation in 2% cysteine solution for 5 min, followed by washes with distilled water and 0.1X Barth solution, as previously described (Slater and Larraín, 2021). Embryos were maintained in glass dishes at 23 °C until reaching the desired Nieuwkoop and Faber (NF) developmental stages (Zahn et al., 2022). All animal procedures were approved by the Bioethical and Biosafety Committee of Universidad San Sebastián.

### Whole-mount immunofluorescence

Embryos at NF35 and NF42 stages were fixed in 4% paraformaldehyde (PFA) for 2 h at room temperature. Samples were then washed with 1X phosphate-buffered saline (PBS). Embryos were permeabilized in a solution containing 1% Triton X-100, 1% DMSO, and 1% bovine serum albumin (BSA) in PBS for 24 h at 4 °C with gentle agitation. Blocking was performed for an additional 24 h at 4 °C using 10% goat or donkey serum in the same permeabilization solution. Primary antibody incubation was carried out for 4 days at 4 °C in blocking solution using the following antibodies and dilutions: anti–TnC (#AB19013) Merck (rabbit polyclonal, AB_2256033, 1:200); anti–MF-20 hybridoma bank (mouse monoclonal, AB_2147781, 1:100); anti–Par3 (H-103) Santa Cruz Biotechnology (rabbit polyclonal, 1:100); anti–E-cadherin clone 5D3 hybridoma bank (mouse monoclonal, AB_528116, 1:100); anti–Isl1 clone 39.4D5 hybridoma bank (mouse monoclonal, AB_2314683, 1:200), Collagen 1 clone SP1.D8 hybridoma bank (mouse monoclonal, AB_528438,1:100) and β1-integrin clone 8C8 hybridoma bank (mouse monoclonal, AB_528309, 1:100). After three consecutive washes with 1% Triton X-100 (20 min each), embryos were incubated for 48 h at 4 °C with fluorescent secondary antibodies Donkey anti-Mouse IgG (H + L), Alexa Fluor 555 (#31570) Invitrogen (AB_2536180, 1:300) and Donkey anti-Rabbit IgG (H + L), Alexa Fluor 647 (#31573) Invitrogen (AB_2536183, 1:300), and protected from light. Nuclear staining was performed using Hoechst or DAPI dye (1:1000). Final washes with 1% Triton X-100 were performed for 24 h.

Embryos were embedded in 1% agarose in distilled water. Optical clearing was achieved using a graded methanol series (25%, 50%, 75%, and 100%; 1 h each), followed by incubation in 50% and then 100% benzyl alcohol/benzyl benzoate (BABB) for 20 min each. Cleared samples were imaged using confocal microscopy, Leica DMI8, or light-sheet microscopy, ZEISS Lightsheet 7.

### Protein extraction

Embryos at stages 14, 30, 35 and 42 were lysed in 50 μL of RIPA buffer per embryo supplemented with protease inhibitors (Sigma-Aldrich #P8340) for total protein extraction. Embryos were mechanically homogenized using a sterile plastic pestle and centrifuged at 4 °C for 20 min at maximum speed. The supernatant was carefully collected, avoiding both the pellet and the upper lipid layer. Protein concentration was determined using the Pierce™ BCA Protein Assay Micro Kit (Thermo Scientific, #23225), following the manufacturer’s instructions. Measurements were performed in triplicate on 96-well plates.

### Western blot analysis

Proteins were separated by SDS–PAGE using 7.5% polyacrylamide gels and transferred onto nitrocellulose membranes. Membranes were blocked with 5% non-fat milk or BSA in TBS-T and incubated with primary antibodies overnight at 4 °C. After washing, membranes were incubated with HRP-conjugated secondary antibodies for 1 h at room temperature. Protein detection was performed using the SuperSignal™ West Pico PLUS chemiluminescent substrate (Thermo Scientific, #34580), and images were acquired with an iBright Imaging System 1500 (Invitrogen).

### Morpholino microinjection

Two antisense morpholinos (MOs) targeting *Xenopus laevis* Fn1 (Fn1-MO) were used, each directed against one of the Fns pseudoalleles (S and L), as both are expressed during *X. laevis* embryogenesis (DeSimone et al., 1992). They were synthesized by Gene Tools, LLC (Philomath, OR), based on previously published and functionally validated sequences (Davidson et al., 2006). The used MO sequences were: XFN1.MO, 5ʹ-CGC​TCT​GGA​GAC​TAT​AAA​AGC​CAA​T-3ʹ; XFN2.MO, 5ʹ-CGC​ATT​TTT​CAA​ACG​CTC​TGA​AGA​C-3ʹ.

Embryos at the 4-cell stage were injected with 10 or 20 ng per cell of a 1:1 MO mixture, as previously described (Davidson et al., 2006). A standard control MO (C-MO) 5′-CCT​CTT​ACC​TCA​GTT​ACA​ATT​TAT​A-3.’ was used as a negative control. Embryos were maintained in 0.1X MMR supplemented with 5% Ficoll during microinjection and for 2 h thereafter. Subsequently, embryos were transferred to 0.1X MMR and cultured at 23 °C until the desired developmental stage was reached.

### Bioinformatic analysis

Protein sequences of the two *Xenopus laevis* TnC homeologs (Tnc.L, UniProt ID: A0A8J0TG81; Tnc.S, UniProt ID: A0A8J0TQA1) and the *Mus musculus* ortholog (TENA_MOUSE, UniProt ID: Q80YX1) were retrieved from UniProt in FASTA format. Multiple sequence alignment was performed using Clustal Omega with default parameters. Conserved motifs and modular domains were identified using ScanProsite, and the results were compared with available structural annotations in UniProt.

### Image analysis

Image processing and three-dimensional reconstruction were performed using Imaris Viewer. Morphometric measurements, including area and circularity, and digital sectioning of regions of interest, were performed in ImageJ/Fiji. Circularity was calculated as 4 *π* × Area/Perimeter ^2^, where a value of 1 corresponds to a perfect circle. Final figures and schematic representations were generated using Adobe Illustrator.

Fluorescence intensity profiles were obtained using line-scan analysis in ImageJ and processed in GraphPad Prism. Each individual profile was normalized separately using min–max normalization, with the smallest value set to 0% and the largest to 100%. Data are presented as a percentage of normalized intensity.

Quantitative fluorescence intensity was measured in defined regions of interest (ROIs) within the DPW. Raw intensity values were normalized to the corresponding DAPI signal to account for differences in image acquisitions.

### Statistical analysis

Statistical analyses were performed using GraphPad Prism. An unpaired t-test was applied for comparisons between two groups, while one-way ANOVA was used for multiple comparisons. A p-value <0.05 was considered statistically significant. Data are presented as mean ± standard deviation of the mean (SD) of at least 3 independent embryos or experiments.

## Results

### DPW undergoes stage-dependent expansion and cellular stratification in *Xenopus laevis*

**FIGURE 1:**
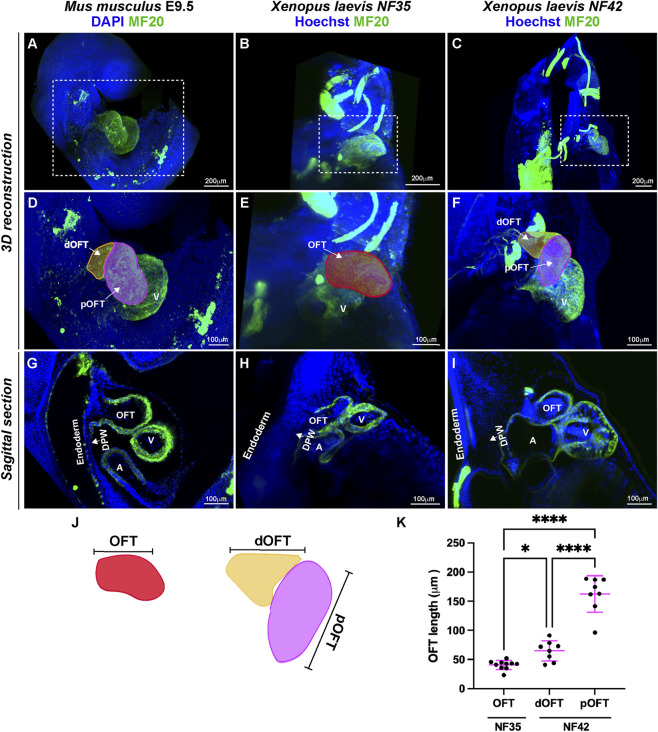
Comparison of the OFT elongation during cardiac development in *Mus musculus* and *Xenopus laevis*. **(A–I)** Three-dimensional (3D) reconstructions **(A–C)**, magnified views of the boxed regions **(D–F)**, and representative sagittal sections **(G–I)** of the embryonic heart in *Mus musculus* at E9.5 and in *Xenopus laevis* at NF35 and NF42 stages. Nuclei were labeled with DAPI or Hoechst (blue), and myocardium was visualized with MF20 (green). In *Mus musculus* E9.5 embryos **(D)**, white arrows indicate the pOFT and dOFT regions of the OFT. In *Xenopus laevis*, comparable cardiac structures are observed at NF42 (F). Sagittal sections highlight the relative arrangement of the OFT, V, and A. Abbreviations: V, ventricle; A, atrium; OFT, outflow tract (red); dOFT, distal outflow tract (yellow); pOFT, proximal outflow tract (purple); DPW, dorsal pericardial wall. **(J)** Schematic representation of OFT and measurement strategy. At NF35, the OFT was quantified as a single undifferentiated domain, whereas at NF42, dOFT and pOFT regions were measured separately. **(K)** Quantification of OFT length at NF35 and NF42. At NF35, the total OFT length was measured; at NF42, the distal and proximal segments were quantified independently. Each dot represents one embryo (n = 8–9 embryos per stage). Data are presented as mean ± SD. Statistical significance was determined using one-way ANOVA with post hoc comparisons (*p < 0.05; ns, not significant).

The OFT undergoes complex morphogenetic remodeling during early heart development, prosses that are frequently affected in congenital heart defects. Because early cardiac development in mammals occurs *in utero*, direct observation is technically challenging. Therefore, we compared heart development in *Mus musculus* and *Xenopus laevis*, which undergoes external embryonic development and allows direct access to early stages. To compare cardiac OFT morphology across species, we analyzed the three-dimensional organization of the embryonic heart in mouse embryos at E9.5 and in *Xenopus laevis* at NF35 and NF42, stages at which heart development has been previously described (Kolker et al., 2000; Mohun et al., 2000), using the myocardial marker MF20 (Bader et al., 1982), and 3D reconstruction (Figure 1). MF20 immunostaining showed a well-defined ventricle and OFT in both species, with comparable spatial organization along the proximal–distal axis. In mouse embryos at E9.5, the OFT is clearly subdivided into proximal and distal regions, whereas in *Xenopus* anatomically equivalent OFT structures were observed in all the embryos at NF42, while this subdivision was not observed at NF35 (Figures 1A–F, Supplementary Material Movies S1,S2). Sagittal sections further confirmed the conserved arrangement of the OFT relative to the ventricle and atrium, as well as their spatial localization adjacent to pharyngeal endodermal and mesodermal tissues (Figures 1G–I, Supplementary Material Movies S1,S2). Quantitative analyses further showed that the OFT undergoes significant elongation between NF35 and NF42 (Figures 1J,K).

Notably, Hoechst nuclear labeling revealed that at NF35 the DPW appeared to comprise predominantly a single cellular layer, resembling the organization observed in mouse embryos (Figures 1G,H, white arrows). In contrast, at NF42, the DPW displayed multiple apparent cell layers, suggesting a more complex tissue architecture at later stages (Figure 1I, white arrows).

**FIGURE 2:**
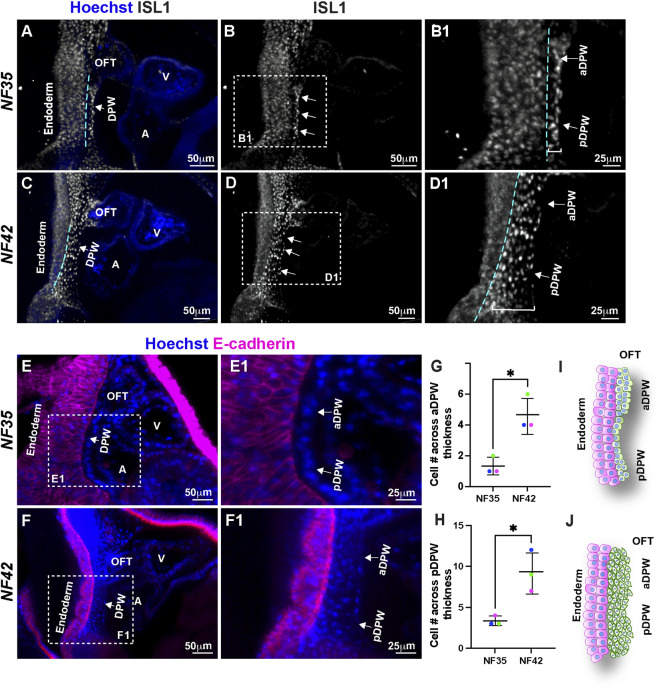
Stage-dependent stratification of the DPW during *Xenopus laevis* heart development. **(A–D1)** *Xenopus laevis* embryos at NF35 **(A–B1)** and NF42 **(C–D1)** were processed by whole-mount immunostaining to detect ISL1 (white) and nuclei (blue). The regions outlined in **(B–D)** are shown at higher magnification in **(B1,D1)**, white arrows indicate the aDPW and pDPW regions. Cyan dashed lines indicate the boundary between endoderm and DPW. DPW thickness is indicated by a white bracket **(E–F1)**. NF35 **(E–E1)** and NF42 **(F–F1)** embryos were processed by whole-mount immunostaining to detect E-cadherin (magenta) and nuclei (blue). The boxed regions in **(E,F)** are shown at higher magnifications in **(E1,F1)**, white arrows indicate the aDPW and pDPW regions. **(G,F)** Quantification of ISL1^+^ nuclei across the thickness of the aDPW **(G)** and pDPW **(H)** at NF35 and NF42. Each dot represents an independent embryo (n = 3 embryos at NF35 and 3 embryos at NF42). Data are presented as mean ± SD. Statistical significance was determined using t-test. **(I,J)** Schematic representations summarizing the stage-dependent transition of the DPW from a predominantly monolayered organization at NF35 **(I)** to a multilayered structure at NF42 **(J)**, while maintaining its spatial relationship with the endoderm and the developing OFT. Abbreviations: V, ventricle; A, atrium; OFT, outflow tract; dOFT, distal outflow tract; pOFT, proximal outflow tract; aDPW, anterior dorsal pericardial wall; pDPW, posterior dorsal pericardial wall.

In mouse embryos, SHF progenitors reside within the DPW, from which they are progressively incorporated into the elongating OFT (Cai et al., 2003; Kelly et al., 2001; Waldo et al., 2005). Given the stage-dependent differences observed in DPW cellular organization in *Xenopus laevis*, we next sought to further characterize DPW cells at NF35 and NF42, with particular emphasis on assessing whether these cells are consistent with a SHF progenitor population. To this end, we performed whole-mount immunostaining to ISL1, a marker of SHF progenitor cells (Cai et al., 2003), and E-cadherin to delineate the adjacent pharyngeal endoderm epithelium (Cortes et al., 2018; Alfano et al., 2019) (Figure 2). At NF35, ISL1^+^ nuclei were detected both in the endoderm and within the DPW domain predominantly organized as a single cellular layer positioned between the endoderm and the developing heart (Figures 2A–B1). In contrast, at NF42 the DPW exhibited increased cellular stratification, with nuclei arranged in multiple layers across the tissue and a noticeable increase in DPW thickness. This change was accompanied by a higher number of ISL1^+^ cells within this expanded region (Figures 2C–D1). E-cadherin labeling consistently marked the pharyngeal endoderm at both stages, indicating that the observed increase in cellular layering is consistent with an expansion of ISL1^+^ cells within the DPW (Figures 2E‐F1).

To further characterize changes in DPW cellular organization across stages and to facilitate comparison with mammalian development, we quantified the number of ISL1^+^ nuclei across the thickness of the DPW at NF35 and NF42. Because the anterior and posterior domains of the DPW contribute to distinct regions of the developing OFT (Bertrand et al., 2011; Francou et al., 2013), these domains were analyzed separately. We observed a significant increase in the number of ISL1^+^ nuclei across the thickness of the aDPW at NF42 compared with NF35 (Figure 2G). A similar increase was detected in the pDPW, where NF42 embryos also exhibited a marked rise in the number of nuclei relative to NF35 (Figure 2H). These observations suggest a progressive expansion and redistribution of ISL1^+^ cells within the DPW between NF35 and NF42.

Together, these results suggest that in *Xenopus laevis*, ISL1^+^ cells localized in the DPW undergo a stage-dependent transition from a predominantly monolayered organization at NF35 to a multilayered structure at NF42, while preserving their spatial relationship with the endoderm and the developing OFT (Figures 2I,J).

### SHF-associated cells in the DPW undergo stage-dependent remodeling of epithelial organization and cell shape during heart development

**FIGURE 3:**
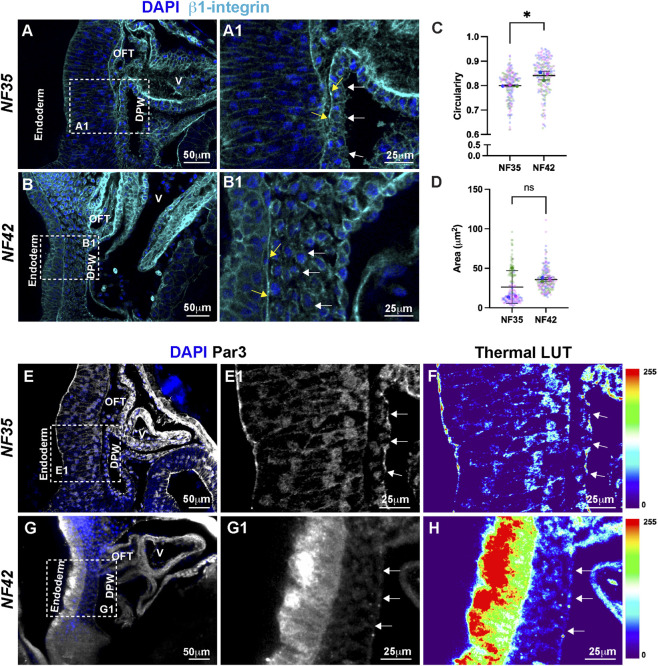
Stage-dependent changes in epithelial organization, cell shape, and polarity of SHF-associated cells within the DPW. **(A,B)** Whole-mount immunostaining of *Xenopus laevis* embryos at NF35 **(A)** and NF42 **(B)**, labeled for β1-integrin (cyan) and nuclei (blue). Dashed boxes in A and B highlight the DPW regions shown at higher magnification in **(A1,B1)** β1-integrin prominently delineates the boundaries of DPW cells (white arrows). Yellow arrows indicate integrin-enriched interfaces facing the pharyngeal endoderm. **(C,D)** Quantification of DPW cell circularity (4πA/P^2^) **(C)** and area **(D)** at NF35 and NF42. Each larger dot in a different color represents the average value for an individual embryo, whereas the smaller dots represent the values of individual cells (n = 50 cells from 3 embryos at NF35 and 3 embryos at NF42). Bars indicate mean ± SD. Statistical significance was determined using t-test. p < 0.05; ns, not significant. **(E–H)** Analysis of epithelial polarity using Par3 immunostaining (white) with DAPI (blue). Representative images at NF35 **(E, E1)** and NF42 **(G,G1)** are shown. Panels F and H display thermal LUT representations of Par3 signal intensity (n = 3 embryos at NF35 and 3 embryos at NF42).

Previous studies in mouse embryos have established that SHF progenitors localized in the DPW exhibit epithelial characteristics that are critical for heart tube elongation, including cell polarity, adhesion, and tissue-level mechanical properties (Francou et al., 2014; Francou et al., 2017; Cortes et al., 2018). Since we observed a stage-dependent increase in cellular stratification of SHF-associated cells in the *Xenopus laevis* DPW (Figure 2), we next asked whether this transition was accompanied by changes in epithelial organization and cell shape. To address this, we performed whole-mount immunostaining for β1-integrin. Integrins are α/β heterodimeric transmembrane receptors composed of α and β subunits that mediate cell-ECM interactions (Sun et al., 2016). We observed that β1-integrin was enriched at the interface between the endoderm and the DPW (Figures 3A1–B1, yellow arrows), consistent with previous observations in mouse (Arriagada et al., 2025). Additionally, we used β1-integrin to delineate DPW cell boundaries, thereby enabling assessment of tissue organization and cell morphology. At NF35, β1-integrin labeling showed that SHF cells showed an epithelial-like organization (Figures 3A–A1, white arrows). In contrast, at NF42, DPW cells exhibited increased spacing between adjacent cells compared with NF35 (Figures 3B–B1, white arrows).

To analyze cell-shape parameters, we marked cell boundaries with β1-integrin. Elongated cells exhibit circularity values near 0, whereas round cells exhibit circularity close to 1. Cell circularity was significantly higher at NF42 than at NF35 (Figure 3C), indicating a stage-dependent shift toward a more circular cell shape during DPW remodeling. However, quantitative analysis showed no significant differences in cell area between NF35 and NF42 DPW cells (Figure 3D).

To further examine epithelial organization and polarity in DPW cells, we analyzed the localization of Par3, a key component of the apical polarity complex (Thompson, 2022). At NF35, Par3 exhibited continuous apical membrane enrichment in DPW cells, consistent with a well-organized epithelial architecture (Figures 3E,F, white arrows). In contrast, at NF42, Par3 localization appeared as a more discontinuous enrichment of Par3 (Figures 3G,H, white arrows).

Together, these findings demonstrate that SHF-associated cells in the *Xenopus laevis* DPW undergo stage-dependent epithelial remodeling characterized by changes in cell shape, spacing, and polarity.

### Stage-dependent ECM remodeling accompanies DPW architectural changes during heart development in *Xenopus laevis*

Given that epithelial remodeling of the DPW was accompanied by changes in cell shape, cell–cell spacing, and apico-basal polarity, we next asked whether this cellular reorganization was associated with concomitant changes in the ECM composition surrounding SHF-associated cells. The epithelial architecture and polarity are tightly regulated by cell–ECM interactions, and ECM components provide both structural support and mechanical cues during heart tube elongation (Rozario and DeSimone, 2010; Lockhart et al., 2011; Arriagada et al., 2025). We therefore asked whether DPW maturation is accompanied by stage-dependent modifications in ECM composition and distribution. To address this, we analyzed the temporal expression and spatial distribution of key ECM components implicated in SHF morphogenesis and OFT elongation, including Fn1, TnC. Col I and Laminin (Lam) (Löhler et al., 1984; Yarnitzky and Volk, 1995; Rahkonen et al., 2004; Astrof et al., 2007b; Derrick et al., 2021; Arriagada et al., 2025). Temporal expression was assessed by immunoblotting using total protein extracts obtained from whole embryos, whereas spatial distribution was evaluated by whole-embryo immunofluorescence with region-specific analysis.

**FIGURE 4:**
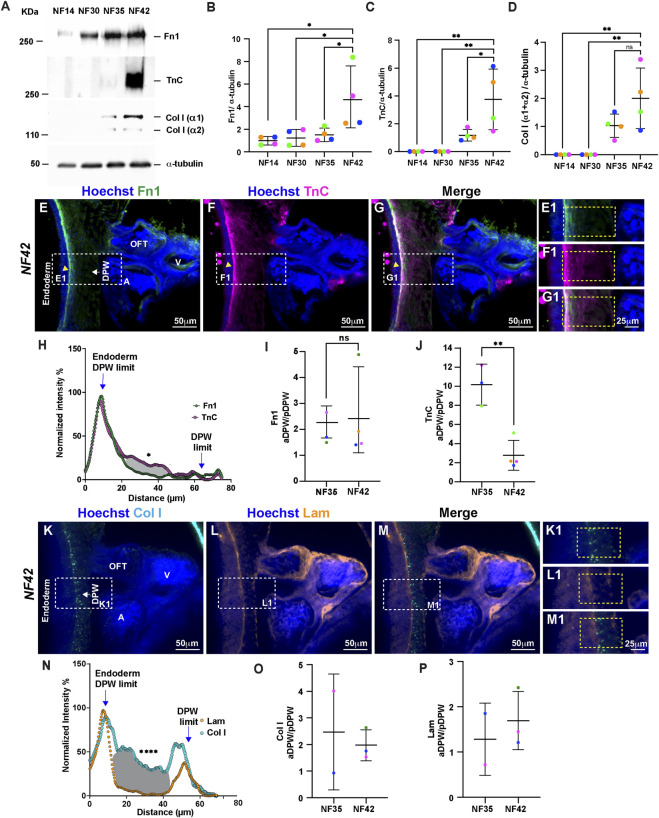
Stage-dependent remodeling of ECM composition and spatial distribution in the DPW during *Xenopus laevis* heart development. **(A)** Representative Western blot analysis of Fn1, TnC, and Col I (α1 and α2 chains) in whole-embryo protein extracts at developmental stages NF14, NF30, NF35, and NF42. α-tubulin was used as a loading control. **(B–D)** Densitometric quantification of Fn1 **(B)**, TnC **(C)**, and Col I (α1+α2) **(D)**, protein levels normalized to α-tubulin. Each dot represents an individual biological replicate (N = 4). Data are presented as mean ± SD. Statistical significance was determined using t-test p < 0.05 (*), p < 0.01 (**), ns: not significant. **(E–G)** Whole-mount immunostaining of NF42 embryos showing Fn1 (green), TnC (magenta), and merged signal, with nuclei labeled with Hoechst (blue). Dashed boxes indicate the DPW region adjacent to the pharyngeal endoderm and OFT. The yellow arrowhead shows Endoderm-DPW limits. **(E1–G1)** Higher-magnification views of boxed regions illustrating Fn1 enrichment along tissue boundaries and TnC localization within the DPW. **(H)** Representative line-scan intensity profiles across the DPW at NF42 comparing normalized Fn1 and TnC fluorescence signals, illustrating their distinct spatial distributions (n = 4 embryos). The analyzed region is indicated by a dashed yellow rectangle in panels E1–G1. **(I,J)** Quantification of fluorescence intensity ratios between anterior and posterior DPW (aDPW/pDPW) for Fn1 **(I)** and TnC **(J)** at NF35 and NF42 (n = 3 embryos at NF35 and 4 embryos at NF42). Data are presented as mean ± SD. Statistical significance was determined using t-test p < 0.05 (*), p < 0.01 (**), ns: not significant. **(K–M)** Whole-mount immunostaining of NF42 embryos showing Col I (cyan), laminin (orange), and merged signal, with nuclei labeled with Hoechst (blue). Dashed boxes indicate the DPW region. **(K1–M1)** Higher-magnification views highlight robust accumulation of Col I surrounding the DPW and a more restricted laminin distribution associated with basement membrane–like structures. **(N)** Representative line-scan intensity profiles across the DPW at NF42 comparing laminin and Col I distributions (n = 3 embryos). The analyzed region is indicated by a dashed yellow rectangle in panels K1–M1. **(O,P)** Quantification of aDPW/pDPW fluorescence intensity ratios for Col I **(O)** and laminin **(P)** at NF35 and NF42 (n = 2 embryos at NF35 and 3 embryos at NF42). Abbreviations: V, ventricle; A, atrium; OFT, outflow tract; DPW, dorsal pericardial wall.

Western blot analysis revealed a progressive, stage-dependent increase in Fn1 protein levels from NF14 to NF42 (Figures 4A,B). In contrast, TnC and Col I were barely detected at early stages (NF14–NF30) and showed a marked upregulation at NF35 and NF42 (Figures 4A,C,D), coinciding with developmental stages at which DPW epithelial organization and cell morphology undergo significant changes. These results suggest that DPW maturation is accompanied by a coordinated temporal remodeling of the ECM.

To assess whether changes in the spatial organization of ECM proteins occur within the DPW, we examined their distribution using whole-mount immunostaining, and performing a line-scan intensity profile in the anterior region of the DPW. Fluorescence intensity profiles were normalized using min–max normalization (0%–100%) for each individual embryo, allowing comparison of spatial patterns.

At NF35, Fn1 and TnC exhibited distinct yet partially overlapping distributions along the DPW–endoderm interface (Supplementary Figure S1). Fn1 was continuously distributed along the DPW, while TnC displayed a more restricted pattern, largely confined to the endoderm and adjacent DPW region. Notably, even though the distribution of TnC is similar to the one observed in mouse, Fn1 distribution differs, as is predominantly localized at the DPW–endoderm interface in mouse embryos (Arriagada et al., 2025).

At NF42, Fn1 remained detectable along tissue boundaries at the DPW–endoderm interface (Figures 4E,E1, yellow arrowhead); however, its intercellular localization within the DPW was markedly reduced in comparison to NF35 (Supplementary Figure S1; Figures 4E–E1). In contrast, TnC exhibited a pronounced redistribution at NF42, with increased accumulation within the endoderm and DPW, and enhanced localization between DPW cells (Supplementary Figure S1; Figures 4F,F1). When we analyzed the distribution of Fn1 and TnC, we observed that they are co-distributed at the endoderm–DPW boundary but display distinct localization patterns within the DPW, with TnC being more prominently enriched between SHF cells than Fn1 (Figure 4G,G1,H). Quantitative analysis of the fluorescence intensity ratio between the anterior and posterior DPW (aDPW/pDPW) reveals that the Fn1 ratio remained largely unchanged (Figure 4I), while there was a significant reduction in the TnC ratio at NF42 compared with NF35 (Figure 4J), indicating a region-specific redistribution.

We next examined fibrillar and basement membrane-associated ECM components at NF42. Col I displayed robust accumulation surrounding the DPW (Figures 4K,K1, M,M1,N), whereas Lam showed a more confined distribution, largely restricted at the DPW–endoderm boundary and the apical region of the DPW (Figures 4L,L1,M,M1,N), with no changes in the aDPW/pDPW distribution (Figures 4O,P).

Together, these data reveal a stage-dependent reorganization of ECM composition within the DPW, characterized by reduced Fn1 and increased TnC intercellular localization at NF42, that temporarily coincides with DPW remodeling and tissue architecture changes during OFT tract development.

Because the localization pattern of TnC in *Xenopus laevis* differs from that described in mouse, we performed an *in silico* comparative analysis of sequence conservation and domain organization across vertebrates (Supplementary Figure S2A). Sequence alignment and domain prediction confirmed that both *Xenopus laevis* homeologs retain the conserved N-terminal assembly domain, EGF-like repeats, constitutive FNIII domains, and the C-terminal fibrinogen-like globe (Supplementary Figure S2B). Differences relative to mouse were restricted to the central FNIII domains (Supplementary Figure S2B, orange domains), which are encoded by alternatively spliced exons and are known to vary across vertebrate species. Despite this variability, the core modular organization of TnC remains conserved. Thus, the differences between the mouse and *Xenopus* TnC sequence are within the alternatively spliced cassette rather than the loss of essential structural domains.

### Fn1 depletion impairs OFT elongation and ventricular morphogenesis

Fn1 is a key ECM component that supports embryonic tissue remodeling by regulating cell–matrix adhesion, collective cell behavior, and force transmission through integrin-dependent mechanisms (Hynes, 1986; Pankov and Yamada, 2002). Genetic and functional studies across vertebrate models have demonstrated that Fn1 is essential for processes requiring extensive tissue remodeling, including gastrulation, somitogenesis, and organogenesis (Astrof et al., 2007a; Davidson et al., 2006; George et al., 1993). Additionally, in mouse models, Fn1 has been implicated in the deployment of cardiac progenitors, myocardial differentiation, and OFT elongation (Arriagada et al., 2025). To determine whether Fn1 is required for cardiac morphogenesis in a conserved manner across vertebrates, we examined the effects of Fn1 depletion over OFT and ventricular development in *Xenopus laevis*.

**FIGURE 5:**
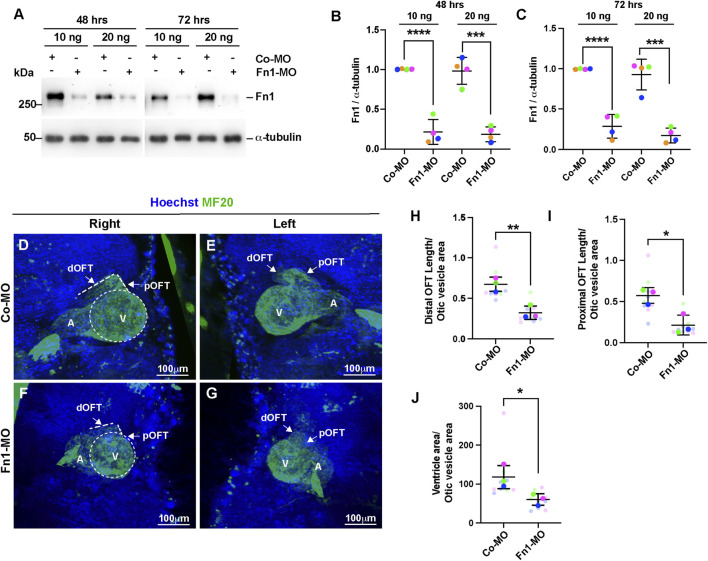
Fn1 depletion impairs OFT elongation and ventricular morphogenesis in *Xenopus laevis*. **(A)** Western blot analysis of Fn1 protein levels in embryos injected with Co-MO or Fn1-MO at 48 and 72 h post-fertilization (hpf). Embryos were injected with 10 or 20 ng of MO, as indicated. α-tubulin was used as a loading control. **(B,C)** Densitometric quantification of Fn1 protein levels normalized to α-tubulin at 48 hpf **(B)** and 72 hpf **(C)**. Each dot represents an individual biological replicate (N = 4). Data are presented as mean ± SD. Statistical significance was determined using t-test. *p* < 0.05 (*), p < 0.01(**), *p* < 0.001(***), p < 0.0001 (****), ns: not significant. **(D–G)** Whole-mount immunostaining for MF20 (green) with nuclear labeling (blue) showing cardiac morphology in control **(D,E)** and Fn1-depleted **(F,G)** embryos at NF42. Representative images of the right **(D,F)** and left **(E,G)** views are shown. Dashed outlines indicate the ventricle, and the arrows mark the dOFT and pOFT regions. **(H,I)** Quantification of distal **(H)** and proximal **(I)** OFT length normalized to the otic vesicle area. **(J)** Quantification of ventricular area normalized to the otic vesicle area. Abbreviations: V, ventricle; A, atrium; OFT, outflow tract; dOFT, distal outflow tract; pOFT, proximal outflow tract. Each larger dot in a different color represents the average for an independent experiment, whereas the smaller dots represent the values of individual embryos (N = 3). Data are presented as mean ± SD. Statistical significance was determined using t-test; *p* < 0.05 (*), p < 0.01(**).

For knocking down Fn1, different concentrations of a 1:1 mixture of previously validated translation-blocking Fn1-MO were injected at the 2-4 cell stage, and Fn1 protein levels were assessed at 48- and 72 hours post‐fertilization (hpf), corresponding to NF35 and NF42, respectively. As expected, Fn1 protein levels were significantly reduced in embryos injected with either 10 or 20 ng of Fn1-MO compared with Co-MO embryos at both 48 and 72 hpf (Figures 5A–C). This reduction confirms an effective decrease in Fn1 expression throughout the period of cardiac morphogenesis.

We next assessed cardiac morphology by whole-mount MF20 immunostaining at NF42. This stage was selected because the OFT is already formed and displays a defined anatomical organization comparable to that described in mouse E9.5 (Kaltenbrun et al., 2011). In contrast, at earlier stages such as NF35, the OFT is still in initial phases of development and lacks a clearly established structure, making it less suitable for assessing OFT morphology and associated ECM organization. Control embryos exhibited a well-extended OFT with clearly distinguishable pOFT and dOFT domains and proper alignment relative to the ventricle and atrium (Figures 5D,E). In contrast, Fn1-depleted embryos displayed a markedly shortened OFT, characterized by reduced extension of both proximal and distal regions and a more compact myocardial configuration (Figures 5F,G), confirmed by quantitative analyses (Figures 5H,I). In addition, Fn1 depletion resulted in a significant decrease in ventricular area (Figure 5J), indicating that Fn1 is required for proper OFT elongation and ventricular morphogenesis. These defects suggest a conserved function of Fn1 during cardiac development.

### Fn1 depletion alters ECM composition during cardiac development

**FIGURE 6:**
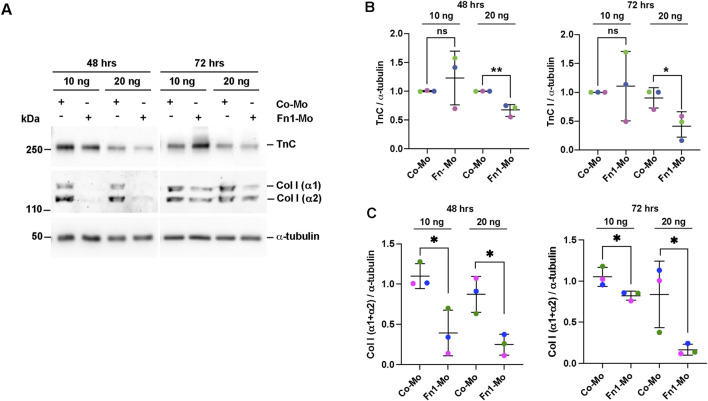
Fn1 depletion alters ECM composition. **(A)** Representative Western blot of TnC and ColI (α1 and α2 chains) protein levels in embryos injected with Co-MO or Fn1-MO at 48 and 72 h post-fertilization (hpf). Embryos were injected with 10 or 20 ng of MO as indicated. α-tubulin was used as a loading control. **(B)** Densitometric quantification of TnC protein levels at 48 hpf (left) and 72 hpf (right), expressed in arbitrary units (a.u.) and normalized to α-tubulin. **(C)** Densitometric quantification of Col I α1+ α2 chain levels normalized to α-tubulin at 48 hpf (left) and 72 hpf (right). Each dot represents an individual biological replicate (N = 3). Data are presented as mean ± SD. Statistical significance was determined using t-tests; *p* < 0.05 (*), p < 0.01 (**); ns, not significant.

Given the established role of Fn1 in organizing the ECM (Sottile and Hocking, 2002), we next asked whether Fn1 depletion affected the abundance and organization of other key ECM components during cardiac morphogenesis. We examined TnC and ColI protein levels at 48 and 72 hpf. Western blot analysis revealed that Fn1 depletion altered ECM protein levels (Figure 6A). TnC protein levels were not significantly changed in Fn1-MO embryos injected with 10 ng; however, a significant reduction in TnC levels was observed at the higher Fn1-MO dose (20 ng) at both time points (Figure 6A,B). In contrast, Col I levels were decreased at both MO concentrations and at both time points evaluated (Figure 6A,C). Together, these data suggest that Fn1 is required for the proper expression of ECM proteins, with Col I appearing more sensitive to Fn1 depletion than TnC.

**FIGURE 7:**
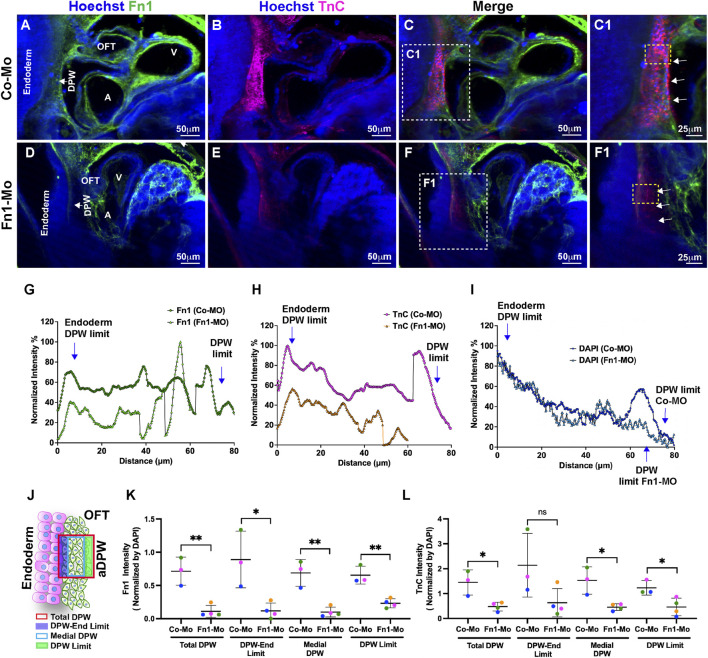
Fn1 depletion decreases TnC expression in the DPW. **(A–F1)** Whole-mount immunofluorescence of NF42 embryos injected with Co-MO **(A–C1)** and Fn1-MO, showing Fn1(green), TnC (magenta), and nuclei labeled with Hoechst (blue). Fn1 and TnC are detected in the DPW, adjacent to the pharyngeal endoderm and the OFT. **(C1,F1)** Higher-magnification views of the boxed regions in **(C,F)**, highlighting intercellular localization of TnC within the DPW (arrows). **(G–I)** Representative line-scan intensity profiles across the DPW illustrating the spatial distribution of Fn1 **(G)**, TnC **(H)**, and DAPI **(I)** in control and Fn1-depleted embryos. (n = 3 Co-MO embryos, 4 Fn1-MO embryos). The analyzed region is indicated by a dashed yellow rectangle in panels **(C1–F1)**. **(J)** Schematic representation of the regions analyzed within the DPW, including total DPW, DPW-endoderm interface (DPW-End Limit), medial DPW, and outer DPW limit. **(K,L)** Quantification of Fn1 **(K)** and TnC **(L)** fluorescence intensity across defined DPW regions, normalized to DAPI signal. Each dot represents a different embryo (n = 3 Co-MO embryos, 4 Fn1-MO embryos). Data are presented as mean ± SD. Statistical significance was determined using t-test, <0.05 (*), p < 0.01(**); ns, not significant.

To assess whether Fn1 depletion alters the spatial organization of TnC within the DPW, we performed whole-mount immunostaining for Fn1 and TnC at NF42, as at this stage the OFT is fully elongated. In control embryos, Fn1 formed a continuous fibrillar network along the DPW–endoderm interface and around the OFT, while TnC displayed a homogeneous signal within the DPW region (Figures 7A–C1, arrows). In contrast, Fn1-depleted embryos showed the expected disruption of the Fn1 fibrillar network together with a marked reduction and discontinuity of TnC signal within the DPW (Figures 7D ‐F1, arrows).

To evaluate changes in spatial distribution independently of absolute intensity, we performed line-scan analysis across the DPW, like in Figure 4. As expected, this analysis revealed that Fn1-depleted embryos exhibit a disruption of the normal spatial pattern in the Fn1 profile. (Figure 7G). Additionally Fn1‐depleted embryos exhibit an altered spatial profile of TnC, characterized by changes in peak distribution and loss of the patterned organization observed in controls (Figure 7H). Additionally, to determine whether these changes were associated with alterations in tissue architecture, we analyzed DAPI distribution along the same axis. Line-scan analysis revealed a reduced spatial extent of DAPI signal in Fn1-depleted embryos compared to controls, indicating a narrowing of the DPW (Figure 7I, arrow). Suggesting that the observed TnC distribution changes reflect the narrowing of the DPW rather than changes in the distribution throughout the tissue.

To quantify regional differences in ECM distribution, the DPW was subdivided into defined domains along its axis, including the DPW endoderm interface, medial DPW, and the DPW limit, as well as the total DPW area (Figure 7J). This approach allowed us to assess whether Fn1 depletion differentially affects specific regions within the tissue.

Fluorescence intensity was quantified in each region and normalized to DAPI to account for differences in cell number and tissue thickness. This analysis revealed a consistent and significant reduction in Fn1 signal across all DPW regions in Fn1-depleted embryos compared to controls (Figure 7K), confirming an overall loss of Fn1 throughout the tissue.

TnC intensity was also significantly reduced in Fn1-depleted embryos across the DPW (Figure 7L). Notably, the extent of reduction varied between regions, with some domains showing more pronounced decreases than others, indicating that Fn1 depletion does not uniformly affect TnC distribution.

Together, these data indicate that Fn1 is required to maintain both normal levels and spatial organization of TnC within the SHF-associated DPW.

### Fn1 depletion reduces DPW area without affecting SHF-associated cells number or individual cell morphology

Given that Fn1 is known not only to organize ECM architecture but also to play a key role in tissue remodeling (Rozario and DeSimone, 2010), we next examined whether Fn1 depletion affects the abundance of ISL1^+^ cells within the analyzed region or the geometry of individual cells within DPW at NF42.

**FIGURE 8:**
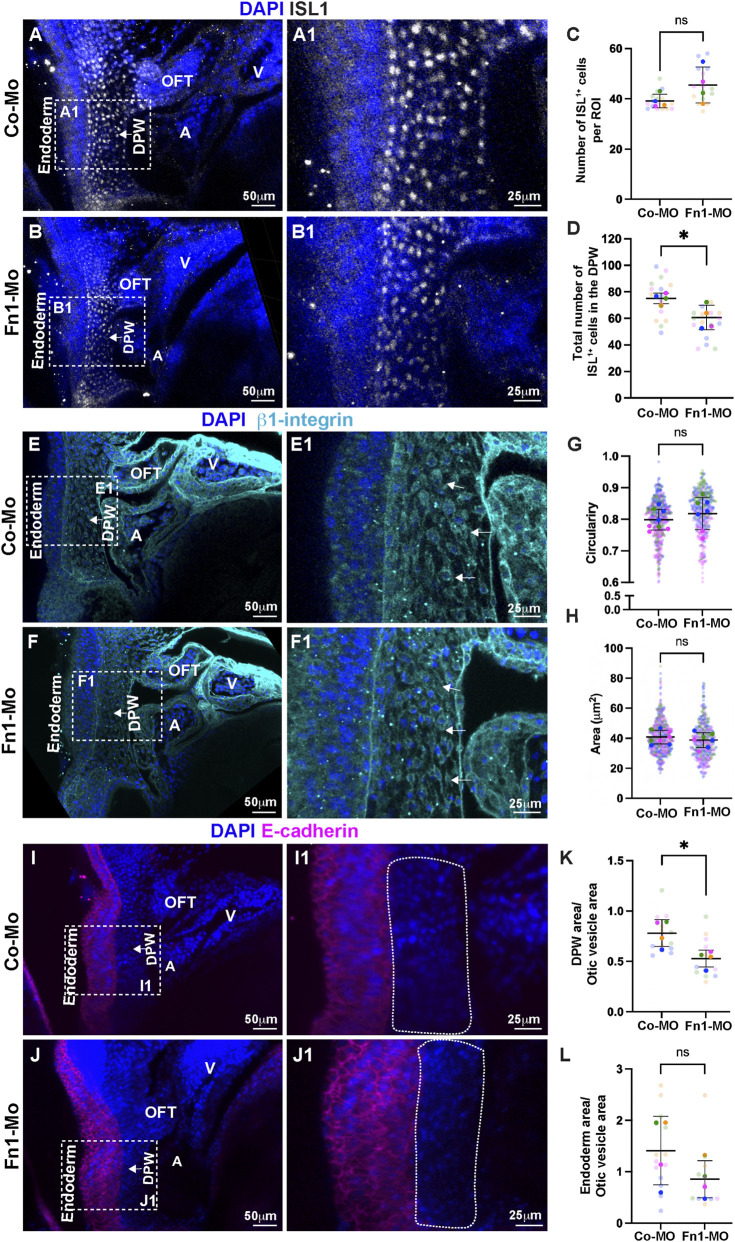
Fn1 depletion reduces DPW tissue area without affecting SHF-associated cells number or individual cell morphology. **(A,B)** Whole-mount immunofluorescence of NF42 embryos injected with Co-MO **(A)** or Fn1-MO **(B)** stained for ISL1 (white) and nuclei labeled with DAPI (blue). Dashed boxes indicate the DPW region located between the pharyngeal endoderm and the developing OFT. **(A1,B1)** Higher-magnification views of the boxed regions showing ISL1^+^ nuclei within the DPW. **(C,D)** Quantification of the number of ISL1^+^ nuclei within a specific ROI and within the whole DPW. **(E,F)** Whole-mount immunofluorescence of NF42 embryos stained for β1-integrin (cyan) and DAPI (blue) in control **(D)** and Fn1-depleted **(E)** embryos. Arrows indicate β1-integrin labeling at DPW cell boundaries. n = 4, different slices were evaluated in 4 Co-MO and 4 Fn1-MO embryos. **(E1,F1)** Higher-magnification views of the boxed regions. **(G,H)** Quantification of DPW cell circularity **(G)** and cell area **(H)** based on β1-integrin-defined cell boundaries. A total of N = 3 independent experiments were performed, each analyzing 2–3 different control (Co-Mo) and Fn1-depleted (Fn1-Mo) embryos. **(I,J)** Whole-mount immunofluorescence of NF42 embryos stained for E-cadherin (magenta) and DAPI (blue) to delineate the pharyngeal endoderm and define DPW boundaries in control **(I)** and Fn1-depleted **(J)** embryos. **(I1,J1)** Higher-magnification views of the boxed regions illustrating DPW morphology. **(K–L)** Quantification of DPW area **(K)** and endodermal area **(L)**, normalized to the otic vesicle area. A total of N = 4 independent experiments were performed, each analyzing 3–4 different control (Co-Mo) and Fn1-depleted (Fn1-Mo) embryos Data are presented as mean ± SD. Statistical significance was determined using t-tests; *p* < 0.05 (*); ns, not significant.

We performed whole-mount immunostaining for ISL1 in control and Fn1 MO embryos (Figures 8A–B1). ISL1^+^ nuclei were detected in the DPW in both conditions and remained spatially associated with the OFT and surrounding tissues. Quantification of ISL1^+^ cells was performed within a defined region of interest (ROI) of fixed area, enabling comparison of local cell density between conditions. This analysis revealed no significant difference in the number of ISL1^+^ cells within the ROI between control and Fn1-depleted embryos (Figure 8C), indicating that Fn1 depletion does not affect the local density of ISL1^+^ cells at this stage.

In contrast, quantification across the entire DPW revealed a significant reduction in the total number of ISL1^+^ cells in Fn1-depleted embryos (Figure 8D). Together, these results indicate that while local cell density is preserved, the overall progenitor pool is reduced in association with an apparent decrease in the DPW region.

To determine if there was a change in individual cell morphology, we analyzed DPW cells using β1-integrin immunostaining to delineate cell boundaries (Figures 8E–F1). At NF42, β1-integrin continued to mark DPW cell membranes in both control and Fn1-depleted embryos. Quantitative measurements revealed no significant differences in cell circularity (Figure 8G) or cell area (Figure 8H) between conditions, indicating that Fn1 loss does not appreciably alter individual DPW cell shape or size.

Because quantitative observations showed a decrease in the overall number of ISL1^+^ cells in Fn1-depleted embryos, we next determined whether this reflected changes in tissue organization. We analyzed DPW architecture using E-cadherin immunostaining to delineate the adjacent pharyngeal endoderm and define DPW boundaries (Figures 8I–J1). Quantitative analysis confirmed a significant decrease in DPW area in Fn1 MO embryos compared with controls (Figure 8K), whereas the endodermal area remained unchanged (Figure 8L). DPW measurements were normalized to the otic vesicle area to account for potential differences in embryo size. Because ISL1^+^ progenitors are located within the DPW, the reduction in DPW area likely reflects a contraction of the ISL1^+^ progenitor domain rather than differences in overall embryo size. Together, these results indicate that Fn1 depletion reduces the size of the SHF progenitor territory by affecting DPW tissue architecture without altering the local density of ISL1^+^ cells.

Together, these data demonstrate that Fn1 depletion does not impair ISL1^+^ cells identity or individual cell geometry but instead reduces DPW tissue size and the total number of ISL1^+^ cells, accompanied by the concurrent loss of key ECM components. This apparent reduction in the total number of ISL1^+^ cells likely reflect the reduced size of the DPW domain rather than changes in local cell density. These findings support the idea that Fn1 is required to maintain proper higher-order DPW architecture during late stages of cardiac OFT development.

## Discussion

### Stage-dependent ECM remodeling accompanies DPW maturation in *Xenopus*

The present work describes, for the first time, the cellular organization of the DPW in amphibians. Our data reveal a clear stage-dependent transition in the organization of ISL1^+^ cells within the DPW between NF35 and NF42. At NF35, the DPW displays a predominantly monolayered organization with relatively few ISL1^+^ cells. By NF42, however, the tissue becomes multilayered and exhibits a marked increase in the number of ISL1^+^ nuclei distributed across its thickness (Figures 1, 2), consistent with the expansion and architectural remodeling of an ISL1^+^ cell population associated with SHF progenitors.

In mammalian embryos, SHF progenitors are typically arranged as a cohesive epithelial layer within the DPW and are progressively incorporated into the elongating OFT rather than forming a multilayered tissue (Francou et al., 2017). Our results indicate that *Xenopus* stages capture distinct and partially uncoupled aspects of these processes. While the degree of OFT elongation observed at NF42 more closely resembles that of mouse embryos at later stages, the overall DPW tissue architecture and spatial organization of ISL1^+^ cells at NF35 more closely resemble the epithelial-like arrangement described in mammalian systems (Kelly et al., 2014).

Together, these observations suggest that *Xenopus* stages do not represent a direct one-to-one equivalent of a single mammalian stage but rather provide a temporal resolution that uncouples processes that occur more concurrently in mammals. This feature allows the identification of dynamic transitions in tissue architecture and ECM organization that may be less readily distinguishable in other models. Importantly, this temporal separation may be particularly advantageous for studying ECM remodeling, as changes in matrix composition and spatial organization can be resolved across developmental stages in which morphogenetic and functional transitions are more discretely distributed.

This architectural transition is accompanied by dynamic changes in ECM composition and spatial organization. Fn1 protein levels progressively increase from NF14 to NF42, as determined by immunoblotting of total protein extracts from whole embryos. However, its spatial organization changes over time. Imaging analyses reveal that at NF35, Fn1 is detected between DPW cells, whereas at NF42 its intercellular localization is reduced. In contrast, TnC shows enhanced intercellular accumulation at NF42, accompanied by increased Col I deposition (Figure 3). These coordinated changes in ECM distribution indicate that DPW remodeling involves reorganization of matrix architecture rather than a simple quantitative increase in ECM abundance.

Our approach focused on a defined set of ECM components with established roles in cardiac development (Fn1, TnC, and Col I) and therefore does not capture the full complexity of the matrix. Future studies using proteomic or transcriptomic approaches will be necessary to systematically characterize ECM composition and directly compare baseline differences between *Xenopus* and mammalian systems.

Importantly, this organization differs from that described in mouse embryos (Arriagada et al., 2025). In the anterior DPW of the mouse, Fn1 is enriched in the mesoderm and supports epithelial cohesion and mechanotransduction. However, ECM components are primarily localized at basal or tissue-boundary interfaces, particularly at the DPW–endoderm boundary, without forming prominent intercellular accumulations. SHF cells, therefore, maintain a tightly packed epithelial arrangement. In contrast, our findings in *Xenopus* reveal substantial intercellular ECM redistribution at later stages, suggesting species-specific differences in matrix topology that may influence epithelial packing and the mechanical environment.

Protein expression levels were assessed using whole-embryo extracts, which do not provide spatial resolution. Therefore, we cannot exclude that the observed changes in ECM protein abundance reflect global alterations in expression rather than DPW-specific regulation. However, the imaging analyses clearly demonstrate a redistribution of ECM components within the DPW, supporting region-specific changes in matrix organization.

Additionally, TnC is known to directly bind to the Fn-1 type III repeats, interfering with co-receptor binding (e.g., Syndecan-4) and preventing the maturation of focal adhesions, thereby reducing cytoskeletal tension (Midwood and Schwarzbauer, 2002). As a consequence, TnC can reduce Fn1-mediated adhesion and generate microenvironments with lower adhesive strength (Radwanska et al., 2017). This transition suggests the possibility of a localized Epithelial-to-Mesenchymal Transition (EMT)-like process, driven by TnC-mediated modulation of the Fn1 scaffold, a mechanism that may facilitate progenitor deployment into the elongating OFT. The increase in TnC between SHF cells may therefore influence the morphology of ISL1^+^ cells, promoting a more rounded morphology, and therefore altering their migratory behavior and reducing epithelial-like organization.

Mechanistically, integrin–Fn1 interactions are key regulators of cell polarity, cytoskeletal organization, and tissue mechanics. Integrin-based adhesions are mechanosensitive structures that couple ECM composition to actomyosin tension and to intracellular signaling pathways such as RhoA and YAP/TAZ (Dupont et al., 2011). In the mouse SHF, mesodermal Fn1 maintains epithelial organization and counteracts TnC-mediated anti-adhesive effects, functioning upstream of mechanotransduction (Arriagada et al., 2025). In *Xenopus*, changes in ECM composition are therefore likely to alter adhesion strength and tissue-level tension within the DPW. Early Fn1 enrichment may provide a pro-adhesive scaffold that promotes epithelial cohesion and controlled progenitor deployment, whereas later ECM remodeling may facilitate decreased cell adhesion, allowing these cells to mobilize and integrate into the elongating OFT. Because integrin signaling dynamically responds to matrix composition, these changes are expected to modulate downstream mechanotransduction pathways, thereby linking ECM remodeling to the expansion and spatial organization of the DPW microenvironment associated with SHF progenitors during OFT elongation.

Because YAP/TAZ signaling is critical for maintaining SHF progenitors in a proliferative, undifferentiated state (Francou et al., 2017). The dynamic ECM remodeling observed here may therefore act as a mechanochemical switch that regulates the balance between progenitor expansion within the DPW and differentiation as cells enter the OFT.

Consistent with this model, it will be important to determine whether DPW remodeling is accompanied by changes in mechanotransduction activity, such as altered YAP/TAZ localization or integrin signaling outputs. Furthermore, the redistribution of ECM components and the apparent loss of epithelial packing raise the possibility that SHF progenitors undergo a partial epithelial-to-mesenchymal transition–like program. Future analyses examining epithelial and mesenchymal marker expression, together with quantitative assessment of progenitor cell morphology and migratory dynamics, will be necessary to determine how ECM remodeling influences SHF cell behavior and deployment during OFT elongation.

### Evolutionary conservation and developmental context of TnC function

Comparative *in silico* analysis of TnC revealed conservation of the N-terminal assembly domain, EGF-like repeats, constitutive FNIII domains, and the C-terminal fibrinogen-like globe (Chiquet-Ehrismann and Tucker, 2011) (Supplementary Figure S2). Differences were restricted to the alternatively spliced FNIII cassette, a highly variable region known to vary across vertebrate species and modulate receptor interactions and matrix-binding properties (Giblin and Midwood, 2015). Thus, the divergent TnC localization in *Xenopus* is unlikely to reflect the loss of structural domains, but rather species-specific differences in splice isoform usage, ECM assembly, or mechanical context.

Alternative splicing of TnC generates multiple isoforms that differ in their interactions with extracellular matrix components and cell-surface receptors. Different splice variants display distinct adhesive properties and differential interactions with Fn1, indicating that TnC biological activity is strongly influenced by its splicing pattern (Ghert et al., 2001; Midwood et al., 2016). In this context, the apparent absence of several FNIII domains observed in our analysis of *Xenopus laevis* may reflect species-specific splice variants that alter TnC interactions with Fn1 and integrins, potentially contributing to the distinct ECM organization observed in the DPW.

Together, these observations suggest that although the core architecture of TnC is evolutionarily conserved, its spatial deployment within the ISL1^+^ cells may be shaped by species-specific regulatory mechanisms controlling splice isoform usage and ECM organization.

### Fn1 depletion reveals its role in ECM assembly within the DPW

To further investigate the role of Fn1 in ECM organization within the DPW, we analyzed embryos depleted for Fn1 using MO antisense oligonucleotides. Consistent with previous studies in mammalian models, Fn1 depletion resulted in cardiac developmental defects, including shortening of the OFT, supporting a conserved role for Fn1in OFT morphogenesis (Figure 5). In addition, these embryos exhibited decreased TnC and Col I protein levels in the DPW (Figure 6). Furthermore, Fn1 depletion also appeared to alter the spatial distribution of TnC within the DPW. In control embryos, TnC is prominently detected between ISL1^+^ cells across the thickness of the DPW (Figure 7). In contrast, in Fn1-depleted embryos, the TnC signal is reduced, more diffusely distributed, with no changes in the distribution.

This pattern suggests that Fn1 networks may be required to maintain proper ECM organization throughout the DPW, particularly in regions of the SHF spatially separated from the endoderm. Consistent with this interpretation, Fn1 fibrils are known to function as structural scaffolds that guide the incorporation and spatial organization of other ECM proteins (Sottile and Hocking, 2002). Accordingly, Fn-1 depletion resulted in decrease TnC and Col I protein levels, and altered TnC distribution, suggesting that Fn1 may act upstream in the assembly and/or stabilization of the matrix within the DPW microenvironment associated with SHF progenitors, contributing to the proper positioning and retention of TnC within the matrix.

Previous studies have shown that Fn1 MO injection in *Xenopus* results in a strong reduction in Fn1 fibril levels during early embryogenesis, although fibril levels can partially recover at later tadpole stages as new protein accumulates (Davidson et al., 2006). Consequently, the phenotypes observed here likely reflect early defects in ECM organization rather than persistent reductions in Fn1 levels throughout all developmental stages.

Because Fn1 depletion was induced at the 4-cell stage, MO-mediated knockdown results in an early and global reduction of Fn1 throughout the embryo. Therefore, the phenotypes observed here reflect a general requirement for Fn1 during early cardiac morphogenesis. In this context, our analysis focuses on the DPW as a defined anatomical and developmental region to examine how this global perturbation impacts ECM organization and SHF cell behavior at the tissue level. These findings support a model in which Fn1 contributes to the establishment of a permissive extracellular environment that enables the correct deployment and spatial organization of SHF progenitors during OFT elongation.

A limitation of this study is that Fn1 depletion was achieved through early-stage, global MO injection, which does not allow spatially restricted manipulation of Fn1 within the DPW. Therefore, we cannot exclude that the observed effects on ECM organization and SHF cell behavior arise from a general reduction in Fn1 levels rather than a DPW-specific requirement. Future studies using spatially or temporally restricted approaches will be necessary to directly address this question.

**FIGURE 9:**
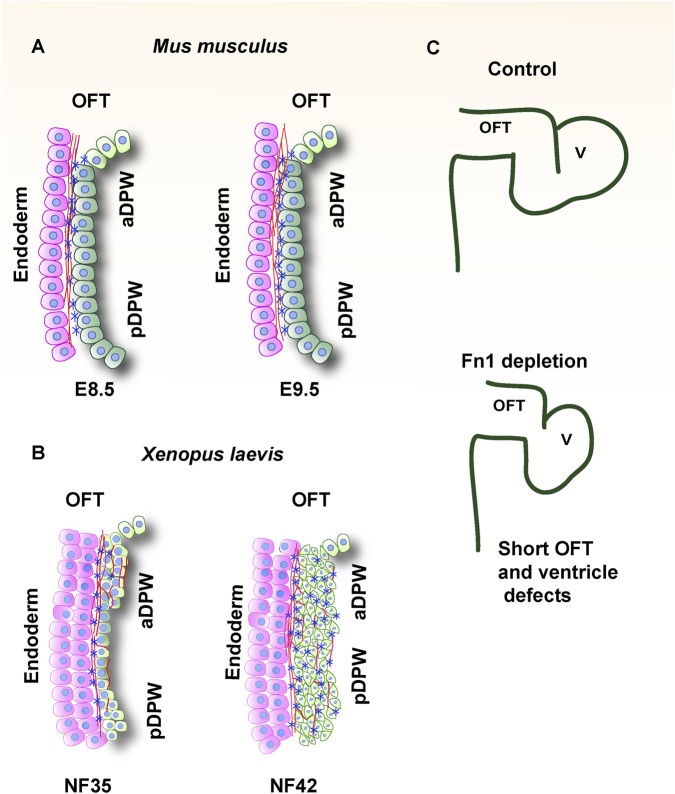
Comparative model of ECM organization in the DPW during OFT development, and the Fn1 role, in *Mus musculus* and *Xenopus laevi*s. **(A)** Model of *Mus musculus* (E8.5–E9.5) DPW organization. Both aDPW and pDPW display a predominantly epithelial organization, with Fn1 (green) and TnC (blue) primarily localized at the interface between the DPW and the adjacent endoderm. **(B)** Model of *Xenopus laevis* (NF35–NF42) DPW organization. DPW undergoes a transition toward a multilayered organization, accompanied by increased intercellular accumulation of Fn1 and TnC within the tissue. **(C)** Model of Fn1 depletion effect over OFT. Functional depletion of Fn1 leads to shortening of the OFT and reduced ventricular size, in both *Mus musculus* and *Xenopus laevis*, highlighting that the requirement of Fn1 for proper cardiac morphogenesis is conserved.

In summary, our study provides the first detailed characterization of the cellular and extracellular architecture of the DPW in amphibians, revealing a highly dynamic, stage-dependent remodeling of the DPW microenvironment associated with SHF progenitors. Unlike the cohesive epithelial organization observed in mammalian models, the *Xenopus* DPW undergoes a pronounced transition into a multilayered structure. This architectural shift is closely coordinated with the spatial reorganization of the extracellular matrix, associated with the interplay between Fn1 and TnC. These differences likely reflect broader evolutionary divergence in cardiac morphology, including the transition from a three-chambered to a four-chambered heart and associated morphogenetic processes. Importantly, despite these structural differences, our findings support the idea that conserved Fn1-dependent mechanisms contribute to OFT elongation, while the specific tissue architecture and morphogenetic strategies are adapted to species-specific cardiac complexity (Figure 9).

Our functional and *in silico* analyses suggest that Fn1 acts as a foundational scaffold required for the proper assembly and retention of TnC and Col I within the DPW. As development proceeds, we propose that species-specific alternative splicing of TnC may enable its intercellular accumulation and modulation of Fn1-mediated adhesion. This process may create a specialized mechanochemical microenvironment, potentially signaling through pathways such as YAP/TAZ, that promotes EMT-like changes in cell morphology and may facilitate the deployment of ISL1^+^ cells associated with SHF progenitors into the elongating OFT.

Although further studies will be required to directly test this model, our findings highlight that while the core molecular components of the cardiac ECM are evolutionarily conserved, their spatial organization can be highly adaptable. Understanding these species-specific differences in matrix architecture and biomechanics not only sheds light on the evolutionary plasticity of heart development but also provides important insights into how dysregulation of ECM scaffolds may contribute to congenital heart defects associated with OFT malformations.

## Data Availability

The raw data supporting the conclusions of this article will be made available by the authors, without undue reservation.

## Supplementary material

SUPPLEMENTARY MATERIAL FIGURE S1 Spatial distribution and co-localization of ECM components in the developing cardiac OFT of *Xenopus laevis* at NF35. **(A,B)** Representative sagittal sections of *Xenopus laevis* embryos showing the distribution of ECM components in the cardiac region. Nuclei were labeled with Hoechst (blue). In **(A)**, Fn1 (green) and TnC (magenta) are detected, while in **(B)**, Col I (cyan) and Lam (orange) are shown. **(A1–A3,B1–B3)** Quantification of normalized fluorescence intensity profiles of Fn1 and TnC along a defined axis across the DPW region. n = 4 different embryos. The region analyzed was indicated by a dashed yellow rectangle in A2-A3. Abbreviations: DPW, dorsal pericardial wall; OFT, outflow tract; V, ventricle; A, atrium.

SUPPLEMENTARY MATERIAL FIGURE S2 Sequence alignment and comparative domain organization of Tenascin-C across *Mus musculus* and *Xenopus laevis*. **(A)** Sequence alignment overview highlighting conserved and variable regions of TnC in *Mus musculus* and *Xenopus laevis* (S and L homeologs). **(B)** Schematic representation of TnC domain organization. The protein consists of N-terminal EGF-like domains (blue and magenta), followed by multiple Fn1 type III (FNIII) repeats (green), including variable FNIII domains (orange), and a C-terminal fibrinogen-related domain (yellow). Comparative analysis indicates conservation of the overall domain architecture across species, with divergence in the number and arrangement of FNIII repeats.

SUPPLEMENTARY MATERIAL MOVIE S1 Cardiac morphology at NF35 in *Xenopus laevis*3D reconstruction of the embryonic heart in *Xenopus laevis* at stage NF35. Nuclei are labeled with Hoechst (blue) and myocardium is visualized using MF20 (green).

SUPPLEMENTARY MATERIAL MOVIE S2 Cardiac morphology at NF42 in *Xenopus laevis*3 D reconstruction of the embryonic heart in *Xenopus laevis* at stage NF42. Nuclei are labeled with Hoechst (blue) and myocardium is visualized using MF20 (green).
