# Supplementary figures and images for: Fibronectin is required for proper extracellular matrix organization and cardiac outflow tract elongation in Xenopus laevis
Source: Front Cell Dev Biol. 2026 May 29;14:1833711. doi: 10.3389/fcell.2026.1833711 (PMC13260624; doi:10.3389/fcell.2026.1833711)

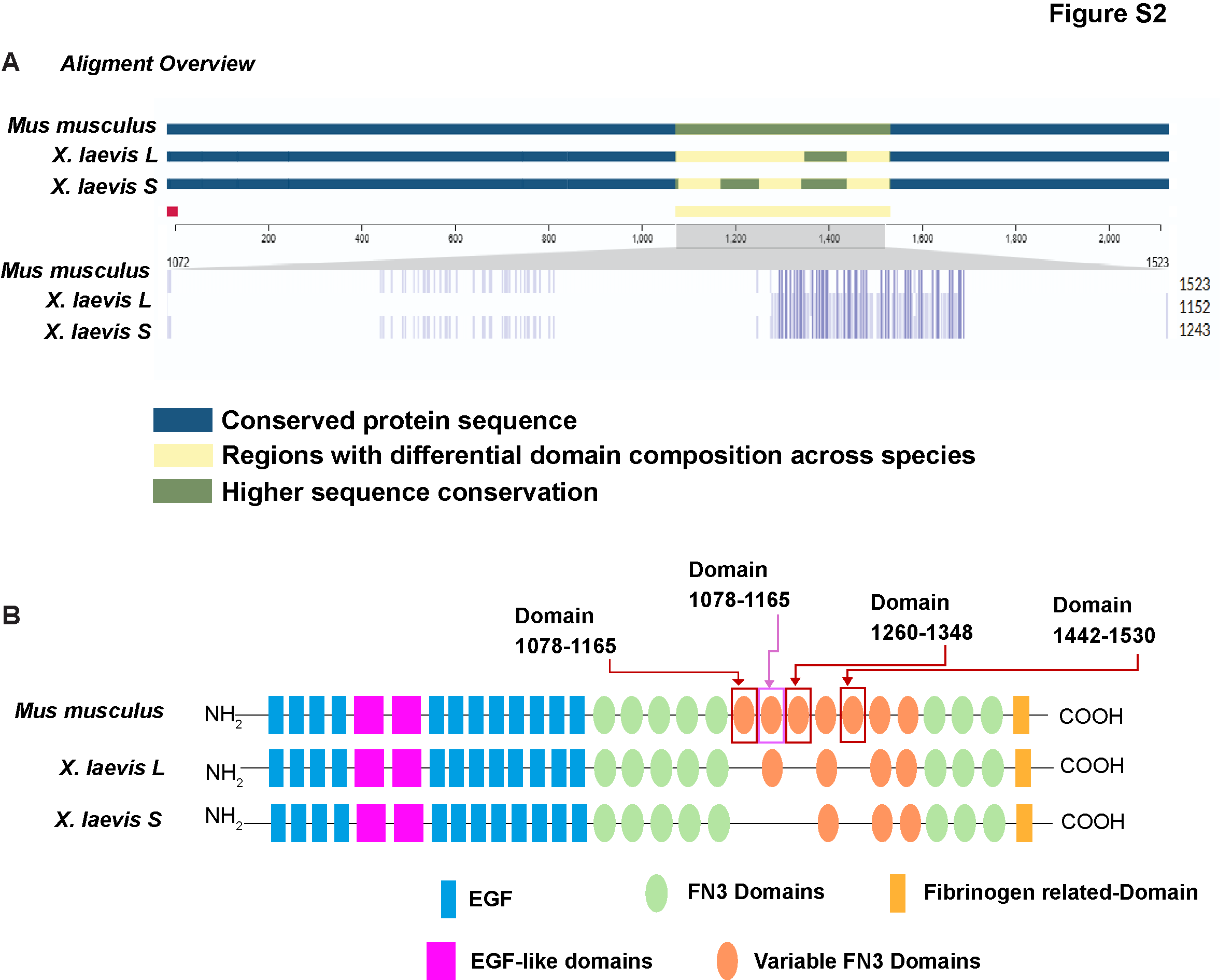

Supplement: Supplementary file 1 [file Image2.tif]

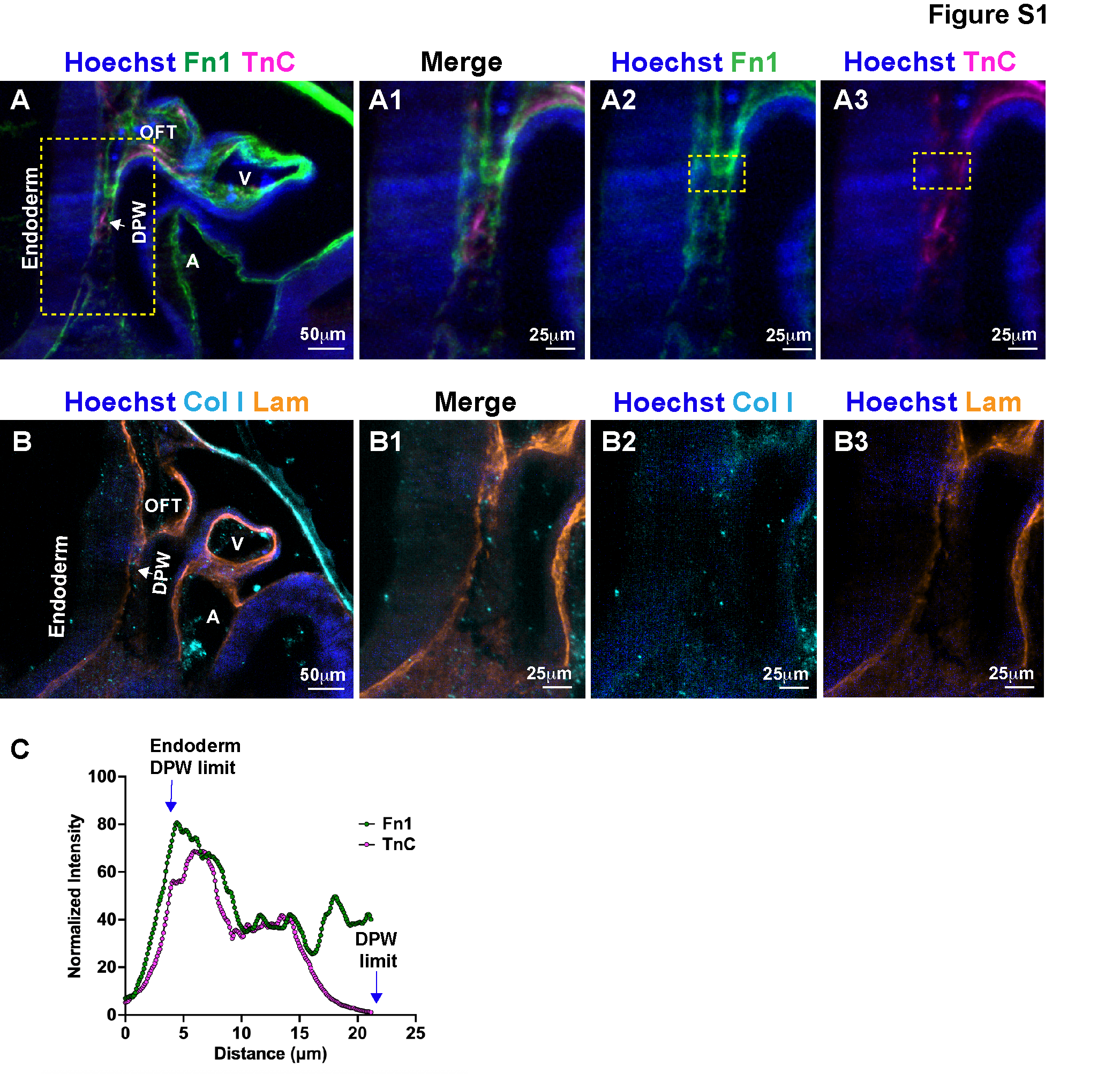

Supplement: Supplementary file 2 [file Image1.tif]
